# Innate Immune Memory in Hematopoietic Stem/Progenitor Cells: Myeloid-Biased Differentiation and the Role of Interferon

**DOI:** 10.3389/fimmu.2021.621333

**Published:** 2021-03-29

**Authors:** Lili Chen, Keiko Ozato

**Affiliations:** Division of Developmental Biology, National Institute of Child Health and Human Development, National Institutes of Health, Bethesda, MD, United States

**Keywords:** HSC, myeloid-bias, trained immunity, epigenetic memory, interferon

## Abstract

Innate immune memory was first described for monocytes and other myeloid cells. This memory is designated *Immune Training*, in which the host animals that had experienced pathogen infection earlier acquire improved resistance to a second infection. Innate immune memory is mediated by an epigenetic mechanism traced to *transcriptional memory* that is conserved throughout evolution and has been selected for the ability to mount an adaptive response to shifting environments. Accumulating evidence shows that not only peripheral myeloid cells but hematopoietic stem/progenitor cells (HSCs/HSPCs) can acquire epigenetic memory upon pathogen exposure. Systemic pathogen infection causes HSCs to exit from quiescence and facilitate myeloid-biased differentiation that leads to efficient host defense. This sequence of events is common in HSC memory generation, which is triggered by different stimuli. Recent studies show that not only pathogens but other stimuli such as metabolic stress can generate memory in HSCs. This review summarizes recent publications relevant to HSC memory. We discuss the current understanding of initial sensors, soluble mediators/cytokines involved in memory formation, including Type I and Type II interferons along with future implications.

## Introduction

Epigenetic traits, such as histone modifications and certain gene expression programs are inherited through somatic cell divisions, allowing for the maintenance of phenotypic attributes across cell generations. The inheritance of epigenetic traits is largely attributed to transcriptional memory, an evolutionarily conserved mechanism known from bacteria to plants, and mammals (1). Typically, in transcriptional memory, certain sets of genes that had been expressed earlier in response to external cues, mount a faster and greater transcriptional response when these genes are induced again. Enhanced transcriptional response in turn provides the capacity to adapt to a shifting environment which can improve survival (1–4). Faster and greater response, however, does not represent the entire range of transcriptional memory, as in some cases, a previous induction renders the gene(s) unresponsive to the subsequent stimulus, illustrating a dual feature of memory. Innate immune memory/trained immunity shares these features.

### Hematopoietic Stem Cells (HSCs)

Adult hematopoietic stem cells (HSCs) reside in the bone marrow (BM) and hierarchically give rise to all lineages of immune cells, which subsequently migrate into peripheral blood and tissues to perform various physiological functions (5–7). HSCs are heterogeneous with respect to self-renewing and differentiation activity (8, 9). Long-term HSCs (LT-HSCs) are capable of self-renewal and full-range lineage differentiation. Short-term HSCs (ST-HSCs) and multipotent progenitors (MPPs) are generated from LT-HSCs. While they maintain multipotency, these progenitors no longer have the self-renewal capacity. MPPs give rise to downstream progenitors, i.e., common lymphoid progenitors (CLPs), common myeloid progenitors (CMPs), megakaryocyte-erythroid progenitors (MEPs), which generate functional lymphocytes and myeloid cells (10, 11).

When encountered with systemic infection, inflammation, blood loss, or other forms of hematopoietic stress, HSCs exit from a dormant state, undergo proliferation, and then differentiation to facilitate efficient myelopoiesis (12, 13). This process is accompanied by peripheral production of hematopoietic growth factors and cytokines, such as granulocyte-macrophage colony-stimulating factor (GM-CSF), Interleukin-6 (IL-6), IL-1, and Type I and Type II interferons (IFNs), which activates new signaling pathways (14–20). Furthermore, HSCs/HSPCs express pattern recognition receptors (PRRs), such as Toll-like receptors (TLRs), and recognize pathogen components, which could then induce cytokines themselves to facilitate emergency myelopoiesis (21–24).

After acute HSC proliferation and myeloid cell differentiation subside, a new homeostasis is established in HSCs which possess a new chromatin landscape and epigenetic traits. This epigenetic modification is thought to provide a basis of innate immune memory/trained immunity, which typically confers enhanced myelopoiesis and greater pathogen clearance (25). Conversely, in other cases, initial priming causes an unresponsive state, resulting in a reduced response upon secondary stimulus, as typified by bacterial lipopolysaccharides (LPS) from gram-negative bacteria (26–29). In either case, innate immune memory is dependent upon epigenetic mechanisms, and as such differs from the classical immunological memory in B and T lymphocytes, which involves genetic changes in the immunoglobulin, and T cell receptor genes, respectively. Unlike adaptive immune memory, innate immune memory created in peripheral myeloid cells is thought to be short-lived, since these cells are turned over relatively rapidly. However, memory in HSCs/HSPCs, if formed, could persist longer, and produce greater downstream consequences.

## Pathogen Component-Induced Innate Immunity

**Figure 1:**
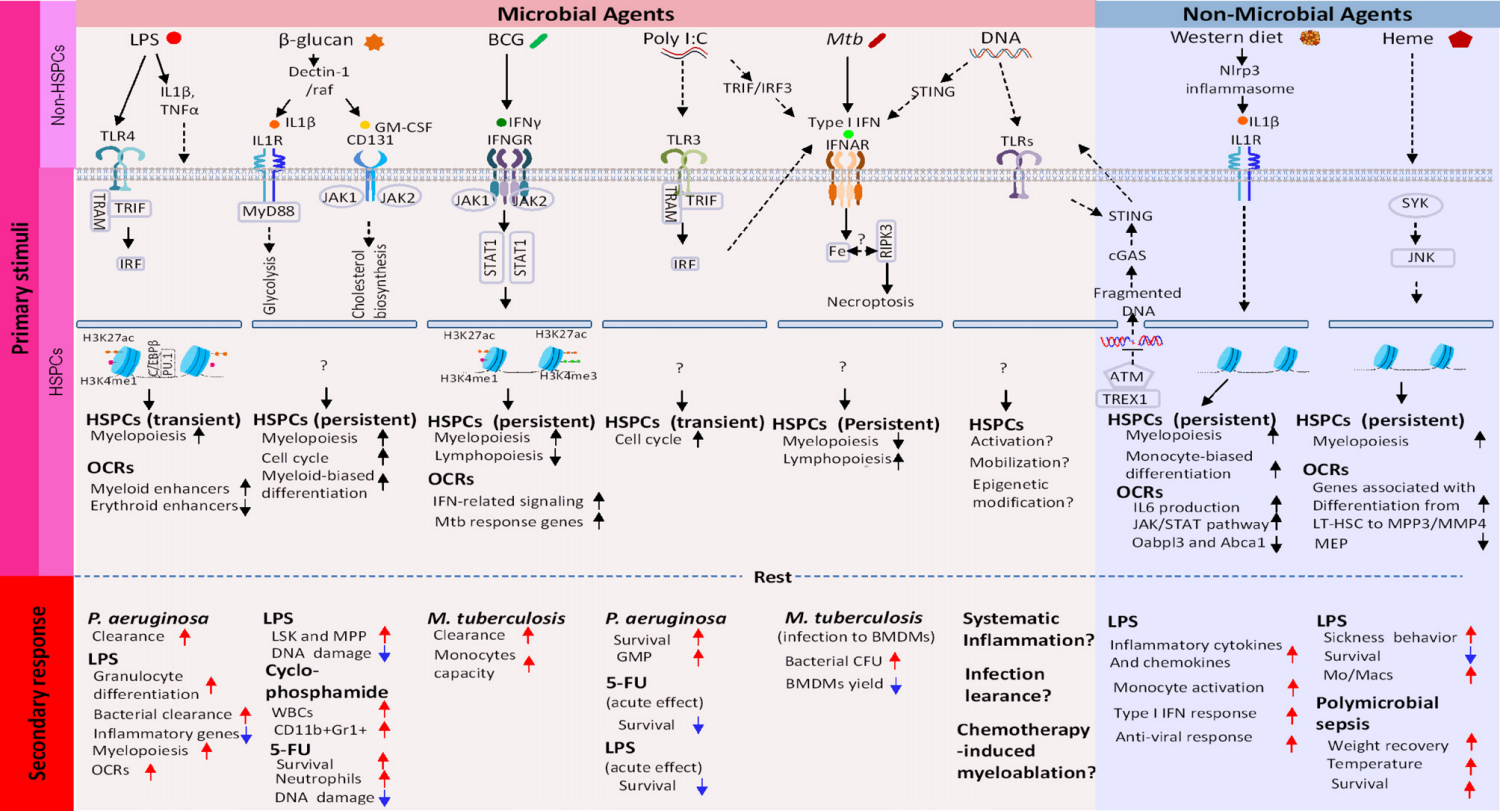
Molecular cascades that create epigenetic memory in HSCs. Top row: Microbial Training Agents and Non-Microbial Training Agents recognized by PRRs and other sensors. Images underneath are subsequent events occurring in descending order. (1): Activation of signaling pathway involving transcription factors and kinases. (2): This then globally alters chromatin accessibility, which leads to building new transcriptome profiles. Open chromatin regions (OCR) can persist longer than transcriptome changes, providing a basis of lasting epigenetic marks. Shown in the bottom two rows are (3): Duration of memory and (4): Phenotypic manifestation of memory. In all cases, HSC memory acquisition involves exit from quiescence, proliferation, and myeloid-biased differentiation of LT-HSC and progenitor cells.

A broad range of microbial infections results in alterations in the BM compartment, involving rapid proliferation and differentiation of HSCs as well as progenitor cells, and the subsequent mobilization to the site of infections (30). *Escherichia coli* infection leads to enhanced granulopoiesis and mobilization of progenitor LK (Lin^-^ckit^+^Sca-1^-^) cells into the peripheral circulation (31). In addition, in the *Pseudomonas aeruginosa* induced sepsis model, the infection causes HSC expansion that permits rapid compensation to cover the loss of mature immune cells (32). Extensive alterations in the HSPCs compartment have also been observed after other forms of systemic infection (33–35). As summarized in **Figure 1**, systemic pathogen exposure can afford improved protection against secondary infection by related or unrelated pathogens (25, 36–40).

### Toll-Like Receptor 4 (TLR4) -Induced HSC Activation and Acquisition of Innate Immune Memory

TLRs (10 in humans, and 13 in mice known) detect pathogen-associated molecular patterns (PAMPs) from invading microbes (23, 24). HSCs/HSPCs express a number of TLRs, including TLR1-4, and TLR 6-9 (41), allowing the cells to recognize various forms of PAMPs and to stimulate proliferation and differentiation into myeloid cells. It is reported that HSPCs (Lin^-^IL-7Rα^-^ckit^+^Sca-1^+^ (LKS+) cells, LKS^+^Flk2^−^ long-term stem cells (LT-HSCs), LKS^+^Flk-2^+^ multipotent progenitors (MPPs) are capable of responding to LPS through TLR4 or Pam3CSK 4 *via* TLR2. The downstream adaptor, MyD88 is shown to be required for HSPC activation (21). Another study, on the other hand, reported that LPS induced TLR4 activation depends on TRIF, an alternate adaptor in the TLR signaling cascade (22). Although seemingly inconsistent, these results may not be contradictory, since TLR4 employs both MyD88 and TRIF (23, 24).

Recently, de Laval et al. reported that upon LPS exposure, HSCs undergo expansion and myeloid differentiation and gaining epigenetic memory, which provided an increased protective response to Gram-negative bacteria, *Pseudomonas aeruaginosa by* reducing bacterial burden and increasing survival rate (38). Although LT-HSC populations returned to a steady-state (cell number) 4 weeks following LPS priming, the LT-HSCs, conferred protection against p. *aeruaginosa* infection when transferred into naïve mice. The LT-HSCs retained the self-renewal and lineage differentiation capacity along with the transcriptome profile of quiescent HSCs, in which LPS induced inflammatory gene expression was transiently seen earlier. LPS induced a number of transcription factors known to promote myelopoiesis, including members of the C/EBP, ATF, and IRF families, which correlated with a sustained change in chromatin accessibility with an increase at PU.1 and RUNX1 motifs. Consistent with this, open chromatin regions correlated with enhancer marks such as H3K3me1 and H3K27ac and are linked to genes involved in myeloid cell development and activity. These observations indicate that LPS induced transcription factors set a new epigenetic mark in chromatin that leads to the establishment of innate immune memory. Accordingly, HSCs without C/EBPβ were unable to alter chromatin accessibility and failed to provide memory. Other transcription factors expressed in HSCs and regulated by LPS may also modulate these processes (42, 43). Thus, persistent alteration of epigenetic landscape is likely to reflect the state and duration of HSC innate immune memory. In line with this study, another paper reported that LPS priming improved bacterial clearance and survival of mice when challenged with *P. aeruginosa* (37). In addition, increased granulocyte monocyte progenitors (GMP) were also found in an LPS mediated sepsis model (38, 44).

Besides these studies, LPS is known to cause a profound unresponsive state known as LPS tolerance after a single administration (26–28). Thus, LPS tolerance can leave the host more vulnerable to a secondary infection in some cases. Tolerance is the opposite side of innate immune memory/trained immunity, in which many proinflammatory cytokines, including IL-1, TNFα, and IL-6 remain uninduced after a second LPS stimulation as observed *in vivo* and *in vitro* (45, 46).

### The Role for TLR3 in HSC Training

Poly (I: C), synthetic ds RNA, used as an RNA virus mimic is a ligand of TLR3 (31–34). de Laval showed that when injected into mice, Poly (I: C), like LPS, led to increased resistance to *P. aeruginosa*, showing that TLR3 signaling activated following RNA virus infection could give rise to trained immunity (38). In addition, Ribes et al. showed that intraperitoneal pre-injection of Poly (I: C) protects mice from the intracranial *E.coli* infection, which is known to cause meningoencephalitis (47). Although this study does not present data for HSCs, it indicates that Poly (I: C) is capable of generating some forms of innate immune memory, as it produced broad effects, including those on NK cell mobility and microglia phagocytic activity. Taken together, given that many RNA viruses are major pathogens that afflict all animals, further investigations are warranted to elucidate TLR3 mediated innate immune training, not only in HSCs but peripheral myeloid cells. In this context, RIG-I and MDA5 that also sense viral dsRNA may also play a role in training (48).

### β-glucan Induced Trained Immunity in HSCs

β-glucans are a group of polysaccharides that represent key components of the skeletal cell wall of fungi (such as *Candida albicans*), bacteria, and some plants (such as grain and seaweed) (49, 50). The ability of β-glucans in modulating immune response has been well established. The first evidence for the role of β-glucan in trained immunity was shown in a study of *C. albicans* infection where preinfection with the fungus protected mice from the second, lethal *C. albicans* infection (36). This protection was dependent on monocytes, not lymphocytes, unraveling a novel training effect in monocyte *in vivo* (36). Cellular response to β-glucans is initiated mostly by the binding to dectin-1, studied extensively in macrophages (50–52). In these studies, the long-term epigenetic reprogramming afforded by *C. albicans* or β-glucans exposure was shown to be mediated by dectin-1 and through the noncanonical Raf-1 pathway (36, 53).

Mitroulis et al. showed that β-glucan, when injected intraperitoneally, induces a dynamic change in the proportion of HSCs and MPPs (39). Gene expression analysis illustrated induction of proliferation and differentiation of LT-HSCs towards myeloid lineage-biased CD41^+^ LT-HSCs subsets, along with an increase in myeloid-biased MPPs. β-glucan injection also led to an increase in CMP, GMP, and granulocytes (Gr1^+^CD11b^+^). The authors performed adoptive transfer of LT-HSCs from β-glucan injected mice into naïve mice and showed that LT-HCSs from β-glucan injected mice provides sustained myelopoiesis. In addition, β-glucan training afforded a protective response to LPS induced DNA damage in HSCs. Also, β-glucan training led to improved resistance to cytotoxic drugs, 5-fluoracil (5-FU), and cyclophosphamide, resulting in a marked increase in the survival rate (54–56). Importantly, the authors found that IL-1β is produced upon β-glucan injection, and this cytokine is responsible for HSC expansion and myeloid biased progenitor differentiation. IL-1 was also responsible for a metabolic shift towards glycolysis. Verifying these results, pharmacological inhibition of IL-1 by IL1RA (anakinra) abrogated HSC expansion, myelopoiesis, metabolic change and immune training. Presumably relevant to these findings, it is reported that SHIP1 signaling is involved in β-glucan induced myeloid cell training, suggesting the role for the phosphatase in this process (57).

Extending the observations of Mitroulis et al., Moorlag et al. recently demonstrated that β-glucan dependent immune training offers a broad anti-pathogen protection, not only in mice, but in human against virulent *Mycobacterium. tuberculosis* (*M.tb)* (58). Human monocytes pre-exposed to β-glucan *in vitro* followed by *M.tb* infection had lower bacterial load than those without β-glucan preexposure. RNA-seq and ChIP seq analyses showed that some IL-1 family cytokines/chemokines were upregulated in β-glucan trained monocytes, which correlated with increased the H3K27ac mark that indicates enhancers. Preinjection of β-glucan in mice conferred longer survival in mice in response to the secondary *M.tb* infection. As reported by Mitroulis et al, β-glucan increased LT-HSCs and myelopoiesis in an IL-1 dependent manner. Corroborating the critical requirement of IL-1 signaling, IL-1RA treatment increased *M.tb* burden in the lung. These reports provide substantive evidence that β-glucan educates HSCs through IL-1 pathways.

### Bacillus Calmette-Guérin (BCG) Induced Trained Immunity in HSCs

BCG vaccine is a live, attenuated strain of *Mycobacterium bovis*, used for protection against *M. tb*. Epidemiological studies on BCG vaccination support its efficacy and the role of innate immunity (59). BCG vaccines are also shown to give cross-protection against different pathogens, even cancers (60). Based on the cross-protective activity of BCG, O’Neill and Netea proposed the possibility that BCG vaccination may be beneficial for boosting host resistance against coronavirus, including Covid-19, pandemic at the time of this writing (61). It is reported that peripheral monocytes acquire trained immunity in volunteers who received BCG vaccine. These monocytes expressed higher levels of proinflammatory cytokines, including IFNγ, TNFα, and IL-6 than those without BCG (62, 63). Also, BCG is reported to provide increased resistance against experimental yellow fever in human monocytes (64), which coincides with a shift towards glycolytic metabolism, important for BCG induced training (65). It is known that HSCs are refractory to direct bacterial infection, including BCG and *Mycobacterium avium*, as HSCs do not take up the bacteria (19, 40, 66, 67).

Kaufmann et al. showed that i.v. injection of BCG in mice causes long term innate immune memory in HSCs, conferring improved resistance to second infection by the virulent *M.tb* (40). The authors found that BCG injection facilitates HSC expansion and development of myeloid lineage dominant multipotent progenitor (MPP3) (19, 40). BM derived macrophages from BCG injected mice gave enhanced protection against *M. tb* compared to those from naïve mice. Moreover, in cell transfer experiments, naive mice given BM HSCs (LKS) from BCG injected mice demonstrated lasting protection against *M.tb*, verifying that memory took place in the HSCs. BCG education of HSCs and enhanced resistance to M. *tb* was dependent on IFNγ (Type II IFN), in which Ifngr*-/-* mice lacking IFNγ signaling failed to provide anti-*M.tb* protection. Bulk and single-cell (sc) RNA-seq revealed that this memory correlated with changes in the transcriptome programs in HSCs and MPPs. At the epigenome-level, the transcriptome profiles were associated with the appearance of key enhancer elements marked by acetylation of H3K27.

More recently, Khan et al. asked if the virulent *M.tb* strain, H37Rv generates trained immunity and report the results startlingly different from those observed with BCG (67). *M.tb* infection by i.v. injection or aerosol, weakened host’s ability to mount resistance against the subsequent *M.tb* infection. The weakened resistance was mediated by Type I IFN signaling, and lasted at least a year. While *M.tb* and BCG both expanded LT-HSCs and MPPs, unlike BCG, *M.tb* suppressed myelopoiesis leading to a dramatic reduction of neutrophils and Ly6C^hi^ monocytes in periphery. RIPK1 dependent necroptosis accounted for the neutrophil deficiency. BM derived macrophages from naïve mice which adoptively received HSCs from *M.tb* infected mice were lower in cell yield and deficient in clearing *M.tb in vitro*. Type I IFN signaling was found critical for the increased susceptibility, as Ifnar1*-/-* mice (lacking type I IFN receptor), but not Ifngr*-/-* mice showed better survival after *M.tb* infection than WT mice and displayed reduced phenotypes. The inhibitory role of type I IFNs in *M.tb* infection is partly in line with some of previous clinical/epidemiological studies. Together, *M.tb* trains HSCs somewhat paradoxically to diminish host’s own innate resistance.

## Non-Microbial Agents Induced Trained Immunity

PRRs recognize not only pathogen-derived molecular patterns, but non-pathogen derived patterns, produced endogenously or exogenously. Some of these can lead to trained immunity with extensive phenotypic changes.

### Western Diet-Induced Trained Immunity

Western-style diet (high calorie, high cholesterol), combined with a sedentary lifestyle are prone to cause obesity, type II diabetes, and other health risks. Inflammation in myeloid cells is a major factor for these health problems. Christ et al. reported that Western diet leads to the generation of trained immunity similar to that produced by pathogens described above (68, 69). The authors examined an atherosclerosis model with Ldlr-/- mice, and found that Western diet prompted the expansion of HSPCs and increased myeloid cell outputs as well as the recruitment of myeloid cells to the site of inflammation. In BM, the proportion of HSPCs, MPPs, and GMPs was increased after consumption of the Western diet. Prolonged myeloid prone changes are associated with low-grade inflammation, also seen in aging. In LPS rechallenge experiments, Western diet-fed mice displayed increased monocyte activation and hyper-inflammation, similar to the reported impacts of innate immune training. Analogous effects were previously reported for rabbits fed with cholesterol-rich diets (70). Supporting an epigenetic mechanism, the Western diet altered overall chromatin accessibility with open chromatin regions associated with IL-6 gene expression and the JAK/STAT pathway activity. Moreover, the NLRP3 inflammasome pathway was involved in Western diet-induced trained immunity, in that the diet-induced effects were reversed in *Ldlr^-/-^/Nlrp3^-/-^* double knock-out mice, which exhibited a reduction in systemic inflammation, excessive hematopoiesis, and reprogramming of GMPs. A prolonged Western diet is known to cause cholesterol overloading in HSCs, leading to an increase in the production of growth factors/cytokines, such as GM-CSF and IL-3. Together these studies suggest that prolonged Western diet promotes formation of immune memory in HSCs. However, underlying processes by which Western diet regulates transcription and epigenome programs in HSCs remain to be elucidated.

### Heme-Induced Trained Immunity in HSCs

Heme, a key prosthetic group of hemoproteins or enzymes, is composed of protoporphyrin IX and a ferrous ion (71). Free heme can accumulate excessively during sterile and infectious hemolysis, including hemolytic anemias, ischemia-reperfusion, and malaria, once heme scavengers are over-saturated (72, 73). Heme accumulation increases oxidative stress and systemic inflammatory response (72). Somewhat paradoxically, sickle-caused heme accumulation provides protective effects against *Plasmodium* Infection, partially through the NR2 2/heme oxygenase-1 (HO-1) pathway (74). Moreover, heme can induce IL-1β production in LPS primed macrophages through activation of NLRP3 inflammasomes (72, 75).

Jentho et al. reported that heme administration increases myeloid-biased LT-HSCs (CD41^+^LT-HSC), and myeloid-biased MPPs (MPP3,Flt3^−^CD48^+^CD150^−^LSK) with a concomitant decrease in lymphoid-biased MPP4 cells (Flt3^+^CD48^+^CD150^−^ LSK) (76). In addition, heme-primed mice were more sensitive to LPS induced acute inflammation, leading to an increase in mortality. Conversely, heme-primed mice showed a protective response to smoldering bacterial sepsis induced by peritoneal contamination and infection. ATAC-seq analysis revealed that heme induces a dramatic change in chromatin accessibility, consistent with myeloid cell-prone development. Heme mediated immune training shared common features with β-glucan driven training, such as upregulated glycolytic metabolism, and enrichment of AP-1 motif in accessible chromatin sites. These findings indicate that labile heme mediates training in LT-HSCs facilitating long-term myelopoiesis with varying outcomes in host defense. It remains to be explored how HSCs sense heme and then reprogram myeloid-biased training *in vivo*.

## Role of IFNs in Innate Immune Memory: IFN Action in LT-HSCs

IL-1 and GM-CSF, cytokines produced by β-glucan priming play a role in HSC immune training (39). IFNs are another class of cytokines that take part in generating innate immune memory in HSCs and peripheral myeloid cells.

### Expression and Function of IFNs

There are three types of IFNs, Type I (IFNα/β), Type II (IFNγ), and Type III (IFNλ). Type I and Type II IFNs are shown to be involved in innate immune memory (see below). However, to date, the role of Type III IFN in memory formation has yet to be deciphered. Type I IFNs are encoded by a cluster of related genes (one *Ifnb* gene, many *Ifna* genes), and synthesized mostly in DCs and macrophages in response to PRR signaling, but other non-immune cells such as fibroblasts and epithelial cells also produce Type I IFNs. Type II IFN is encoded by a single gene and synthesized in NK and T cells in response to cytokines such as IL-12 and TCR activation (77, 78). Type I IFNbinds to the surface receptor, IFNAR, and signals through a JAK-STAT pathway, leading to activation of the STA1/STAT2/IRF9 complex. This prompts transcriptional induction of more than 2,000 IFN stimulated genes (ISGs), which collectively confer anti-viral and anti-microbial activity on the host cells (79). Type II IFN binds IFNGR and signals through a similar, but distinct JAK-STAT pathway which activates STAT1 dimers. Type II IFN also induces over 2,000 ISGs, many overlapping with ISGs induced by Type I IFN (79, 80). Together, these IFNs provide innate resistance against all types of pathogens, from viruses (DNA and RNA viruses) to bacteria, fungi, and even parasites, a trait that distinguishes them from other cytokines (81). There is an extensive crosstalk between IFNs and NF*κ*B induced inflammatory responses. For example, IFNβ is activated not only by IRFs but by NFκB, which in turn creates an IFNβ feedback loop. Thus, ISGs and NFκB-induced factors are often co-expressed during infection and inflammation.

Both Type I and Type II IFNs are involved in regulating HSC activity and play a role in forming innate immune memory (18, 38, 40). It is reported that the injection of IFNα and Poly (I: C), a Type I IFN inducer, prompts LT- HSCs to exit quiescence prompting their proliferation (18). This process is dependent on IFNAR and JAK-STAT1 signaling.

Convincing evidence has been presented for the requirement of IFNγ in BCG mediated HSC immune training: Kaufmann et al. showed that priming mice with BCG trains HSCs to form memory, which provided enhanced protection against *M.tb* and that this training was dependent on IFNGR (40). In addition, IFNγ creates innate immune memory in peripheral myeloid cells (82, 83).

Baldridge et al. showed that injection of recombinant IFNγ activates LT-HSCs, triggering the cell cycle entry, and the subsequent mobilization to the spleen (19, 84). Infection with *Mycobacterium avium* which induces IFNγ also stimulated LT-HSC expansion. A later study by Matatall et al. showed that LCMV infection, also inducing IFNγ, triggered LT-HSC proliferation, and directed myeloid-biased differentiation along with an increased expression of C/EBPβ (85). Furthermore, myeloid-biased HSCs expressed IFNGR at higher levels than lymphoid biased HSCs, thus were more sensitive to IFNγ signaling than lymphoid biased HSCs. The differential IFNGR expression reinforced the selective expansion of myeloid-biased progenitors and their differentiation. Furthermore, IFNγ primed myeloid-biased HSC were preferentially mobilized to periphery upon *Mycobacterium. avium* infection (85). These observations support a significant role of IFNγ for HSC memory and provide a clue to the mechanism of IFNγ action.

### IFN Stimulation Creates Classical Transcriptional Memory

In a separate line of approach, our group reported that Type I and Type II IFNs generate transcriptional memory in somatic cells (83). When NIH 3T3 cells, mouse embryonic fibroblasts, and BM macrophages were treated with IFNβ or IFNγ, respectively in advance, the cells mounted a faster and greater ISG response upon the second IFN stimulation, a typical feature of transcriptional memory. Supporting the biological significance of this memory, IFN pretreatment led to improved protection against EMCV viral infection. This memory was inherited through generations, as the memory response was retained after cycles of fibroblast proliferation, another hallmark of transcriptional memory. Transcriptome analysis revealed that memory has a dual quality. While some ISGs exhibited enhanced transcription, other ISGs became unresponsive (or less responsive) to the second IFN stimulation. A similar dual feature has been documented for LPS induced memory, in that LPS pre-administration enhanced expression of some LPS response genes, but repressed other genes (26–28, 38, 45). GO analysis indicated that this duality has a functional meaning, since ISGs showing enhanced expression in the second response were associated with anti-viral, anti-pathogen responses, whereas ISGs with reduced second response were enriched with terms for cell growth, metabolic regulation, etc, unrelated to host defense. The memory response was accounted for by accelerated recruitment of STAT1 and RNA polymerase II to ISGs for ISGs with the enhanced second response. On the other hand, a block in transcriptional elongation was observed for ISGs tolerized in the second response. Epigenome analysis showed that memory coincides with the deposition of the histone H3.3, a conserved histone variant implicated in memory (86). H3.3 and its specific chaperon HIRA, which is responsible for genic H3.3 deposition are expressed highly in murine adult BM HSCs. Our subsequent study with conditional Hira*-/-* mice demonstrated that HIRA is essential for the generation and maintenance of BM LT-HSCs. In the absence of HIRA, the number of BM LT-HSCs were dramatically reduced, along with the reduction in immediate (MPPs) and downstream progenitors (CMPs, GMPs), leading to a marked paucity in mature, functional myeloid and lymphoid cells. These observations support the possibility that the histone H3.3 and its chaperon HIRA play a substantial role in shaping the development and function of HSCs, and may contribute to their memory formation.

### Trained Immunity and DNA Damage in HSCs: Unsolved Questions

Although a limited number of IFN stimulation can generate trained immunity, repeated IFN exposure is shown to exhaust HSC pools by an internally controlled process, not fully understood (84, 87, 88). Transcription factor families including the IRF family appear to play a role (84, 87). HSC attrition is presumably a result of DNA damage that occurs during HSC proliferation and associated replication stress (88). In addition to IFNs, Poly I: C and LPS are shown to cause DNA damage in HSCs even after a single exposure, as evidenced by phosphorylation of H2AX and nuclear foci formation (39, 88). Similarly, chronic exposure to IL-1, a proinflammatory cytokine involved in β-glucan mediated HSC training is shown to exhaust HSC pools (17). Since HSC activation and resultant proliferation leads to DNA damage, it is possible that HSC training is in some way linked to DNA damage. On the other hand, excessive HSC activation/proliferation may have a negative consequence on HSC’s self-renewal capacity and lifespan. It remains unclear how HSC exhaustion affects immune training and vice versa.

There is evidence suggesting that DNA damage activates another signaling pathway, STING, and influences innate immune memory in HSCs. STING is a cytoplasmic adaptor for a DNA sensing signaling pathway (89). Canonical STING ligands, cyclic di-GMP/AMP are produced by various pathogens, which activate TBK and IRF3, resulting in Type I IFN and ISG induction (86). The STING pathway is functional in LT-HSC since they are activated and mobilized by a canonical STING ligand (90). It is now evident that not only cyclic di-GMP/AMP, but DNA breaks produced by genotoxic, chemotherapy drugs activate the STING pathway (91). STING is also activated in mice defective in DNA repair (92). DNA damage-induced STING pathway is reported to chronically activate ISGs and NFκB mediated inflammatory cytokines in some cell types (91, 92).

It is noteworthy that chronic ISG expression and STING activation is a hallmark of Aicardi-Goutieres Syndromes (AGS) and related retinal vasculopathy with cerebral leukodystrophy (RVCL), which produce complex inflammatory diseases often involving neurological defects (93, 94). It may be of interest to study how HSC DNA damage and immune training intersect with AGS and related chronic inflammatory disorders.

## Concluding Remarks

Innate immune memory is an emerging concept that opened a radically new perspective on infection and inflammation. Convincing evidence has been presented demonstrating that HSCs form epigenetic memory in response to pathogens and other stress, which confers adaptive responses to the subsequent stress upon the host. HSC memory coincides with the induction of proliferation and myeloid-biased progenitor differentiation, the process driven by IFN, IL-1 and other inflammatory cytokines. Many questions regarding molecular mechanisms, signaling pathways, and epigenome landscapes leading to HSC innate immune memory remain to be elucidated further. In addition, relationships between HSC immune training, DNA damage, and hematopoietic aging are subjects of future investigation.

## Author Contributions

KO proposed LC to write a minireview on innate immune memory. The two authors discussed the scope of the task, undertook literature surveys, and outlined the structure of the review. Both authors wrote the manuscript and performed editing. All authors contributed to the article and approved the submitted version.

## Funding

This work was supported by the intramural program of the National Institute of Child Health and Human Development, National Institutes of Health (Project designation: ZIA HD 001310-33).

## Conflict of Interest

The authors declare that the research was conducted in the absence of any commercial or financial relationships that could be construed as a potential conflict of interest.
